# Molecular Imaging of Fibroblast Activation Protein in Response to Cardiac Injury Using [^68^Ga]Ga-DATA^5m^.SA.FAPi

**DOI:** 10.3390/ph18050658

**Published:** 2025-04-29

**Authors:** Victoria Weissenböck, Lukas Weber, Michaela Schlederer, Laura Silva Sousa, Anna Stampfer, Simge Baydar, Thomas Nakuz, Raffaella Calabretta, Ana Isabel Antunes Goncalves, Xiang Li, Frank Rösch, Bruno K. Podesser, Lukas Kenner, Marcus Hacker, Attila Kiss, Cecile Philippe

**Affiliations:** 1 Department of Biomedical Imaging and Image-Guided Therapy, Division of Nuclear Medicine, Medical University of Vienna, 1090 Vienna, Austria; victoria.weissenboeck@meduniwien.ac.at (V.W.);; 2 Center for Biomedical Research and Translational Surgery, Medical University of Vienna, 1090 Vienna, Austria; lukas.weber@meduniwien.ac.at (L.W.); attila.kiss@meduniwien.ac.at (A.K.); 3 Department of Pathology, Medical University of Vienna, 1090 Vienna, Austria; 4 Ludwig Boltzmann Institute for Cardiovascular Research, 1090 Vienna, Austria; 5 Institute of Nuclear Chemistry, Johannes Gutenberg University Mainz, 55128 Mainz, Germany; 6 Ludwig Boltzmann Platform for Comparative Laboratory Animal Pathology, 1090 Vienna, Austria; 7 Unit of Laboratory Animal Pathology, University of Veterinary Medicine, 1210 Vienna, Austria

**Keywords:** FAP, FAPI tracer, cardiac fibrosis, PET

## Abstract

**Background/Objectives:** Fibroblast activation protein (FAP) has gained tremendous traction as a target for tumor imaging and cancer treatment, while also playing a key role in fibrosis. Our study aimed to evaluate [^68^Ga]Ga-DATA^5m^.SA.FAPi for PET imaging of replacement fibrosis following myocardial infarction (MI) or interstitial fibrosis associated with hypertrophy. **Methods**: MI or transverse aortic constriction (TAC)-induced hypertrophy was induced in C57BL/6 mice, with sham-operated animals serving as controls. At multiple time points during disease progression (1, 2, and 6 weeks post-surgery), [^68^Ga]Ga-DATA^5m^.SA.FAPi PET/CT scans were performed, followed by ex vivo investigations. Additionally, in vitro cell uptake experiments simulating hypertrophy were conducted. **Results**: Cardiac uptake of [^68^Ga]Ga-DATA5m.SA.FAPi significantly increased two weeks after MI induction (MI: 2.1 ± 0.2%ID/g, *n* = 7 vs. SHAM: 1.1 ± 0.2%ID/g, *n* = 5; *p* = 0.002), confirmed by ex vivo autoradiography. No significant difference was observed at six weeks post-MI (MI: 1.1 ± 0.1%ID/g, *n* = 4 vs. SHAM: 0.8 ± 0.0%ID/g, *n* = 3), indicating infarct healing completion. In contrast, TAC mice showed increased uptake after six weeks (TAC: 1.8 ± 0.2%ID/g, *n* = 6; *p* = 0.007), related to interstitial fibrosis progression. Consistently, high-stretched cardiac fibroblasts demonstrated a higher uptake compared to low-stretched conditioned ones, suggesting the stretch mediates regulation of FAP. **Conclusions**: This study demonstrated the efficacy of [^68^Ga]Ga-DATA^5m^.SA.FAPi for longitudinal imaging of cardiac fibrosis in response to different cardiac injuries. In vivo FAP imaging during cardiac remodeling may serve as a valuable tool for diagnosing and predicting disease progression, ultimately aiding in the clinical management of patients.

## 1. Introduction

Over the past decade, cancer research has increasingly focused on the tumor microenvironment (TME), with cancer-associated fibroblasts (CAFs) playing a pivotal role in its remodeling. Notably, the fibroblast activation protein (FAP), expressed on the surface of CAFs, has gained significant attention as both a diagnostic and therapeutic target. Non-invasive imaging of FAP via positron emission tomography (PET) has emerged as a promising method for the detection and monitoring of cancer. Since 2018, numerous FAP inhibitors (FAPIs) have been developed as tracers for tumor imaging via PET/computed tomography (CT), labelled with short half-life isotopes such as gallium-68 [1,2,3,4,5] or fluorine-18 [6,7]. Furthermore, indium-111 is also used for imaging purposes [8]. For therapeutic applications, these tracers are labelled with lutetium-177 [3,8] or rhenium-188 [3,9], extending their applications as theranostic agents. Particularly, [^68^Ga]FAPI-04 and [^68^Ga]FAPI-46 have garnered considerable attention due to their potential for imaging various cancer types [3,10,11,12]. In addition, [^68^Ga]FAP-2286 has emerged as a promising tracer among the FAPIs, demonstrating a strong track record in clinical applications [8].

Beyond its expression in the TME, FAP is highly upregulated during fibrotic tissue remodeling, making FAPI tracers attractive tools for visualizing and monitoring fibrotic progression. In 2022, more than 100 million people were affected by organ fibrosis, characterized by fibroblast activation and the excessive growth of connective tissue (collagen formation), which can disrupt the affected organ until complete loss of function [13,14,15]. As a result, approximately 45% of all deaths in industrialized countries are attributed to fibrotic diseases, whereof the majority accounts for lung and cardiac fibrosis [16]. This high prevalence underscores the critical need for advanced diagnostic and therapeutic approaches to effectively treat these life-threatening conditions. In this context, studies have investigated the potential of FAPI-PET-tracers for early detection and monitoring of cardiac fibrosis [2,5,7,11,12,17,18,19,20,21,22,23,24,25,26]. Varashte et al. [2] were the first to study the progression of cardiac fibrosis following acute myocardial infarction (MI) in mice. [^68^Ga]FAPI-04 PET imaging showed a significantly increased uptake within the infarcted area six days after MI insult. Another model of cardiac fibrosis was explored by Wang et al. [17], who demonstrated a significantly increased cardiac uptake with the same tracer eight weeks after inducing pressure overload heart failure. Collectively, these data suggest that FAPI tracers may serve as sensitive biomarkers for cardiac fibrosis and therapeutic targets. However, there is limited evidence on the effectiveness of FAPI tracers for long-term monitoring of cardiac fibrosis. In addition, the comprehensive spatial and temporal expression of FAP in MI-induced replacement versus pressure overload interstitial fibrosis remains poorly understood.

Building on previous investigations [2,17,21,25,26,27], the aim of our study was to further explore the potential of FAP imaging in the context of MI and pressure overload induced left ventricular (LV) hypertrophy. We propose [^68^Ga]Ga-DATA^5m^.SA.FAPi as an alternative to the well-established FAPI tracers, which can be limited in availability. Unlike other FAPI tracers, [^68^Ga]Ga-DATA^5m^.SA.FAPi incorporates a basic squaric acid (SA) motif, which simplifies synthesis by eliminating the need for protecting groups and also demonstrates positive pharmacological effects [4,28]. Moreover, the use of DATA as chelator, as opposed to the conventionally used DOTA, allows for gallium-68 labelling at room temperature in just 20 min. These improvements make [^68^Ga]Ga-DATA^5m^.SA.FAPi a promising candidate for clinical application and further research. We complemented our preclinical imaging study with the investigation of [^68^Ga]Ga-DATA^5m^.SA.FAPi uptake in human myocardial fibroblasts and a qualitative immunohistochemistry (IHC) analysis, which included FAP staining along with additional extra-domain B fibronectin (ED-B FN) staining. ED-B FN, a key component of the extracellular matrix (ECM), is associated with fibrosis and serves as an indicator of cell proliferation and collagen deposition [29]. Furthermore, as part of a prospective clinical study, we imaged a patient with severe aortic stenosis and suspected amyloid light chain (AL) cardiac amyloidosis using this tracer. Hence, our study represents a significant contribution to the molecular imaging of cardiac fibrosis using this novel FAP inhibitor compound, paving the way for further clinical applications and research.

## 2. Results

### 2.1. In Vivo Cardiac Imaging of [^68^Ga]Ga-DATA^5m^.SA.FAPi

**Figure 1:**
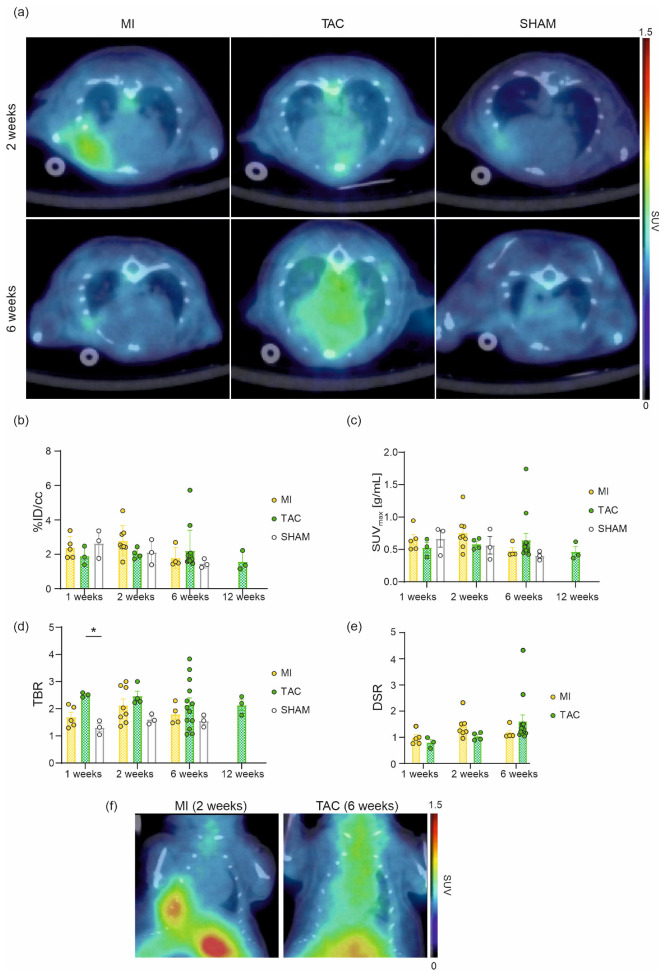
Longitudinal cardiac in vivo PET/CT imaging and quantitative analysis of MI, TAC, and SHAM (control) mice. Representative [^68^Ga]Ga-DATA^5m^.SA.FAPi PET/CT scans (axial view) showing [^68^Ga]Ga-DATA^5m^.SA.FAPi uptake in the infarcted myocardium (MI) 2 weeks post-surgery and homogeneous uptake in the entire heart of the pressure overload hypertrophy mouse model (TAC) 2 and 6 weeks post-surgery (**a**). Uptake quantification of [^68^Ga]Ga-DATA^5m^.SA.FAPi expressed as %ID/cc (**b**), SUV_max_ (**c**), tissue-to-background ratio (TBR) (**d**), and disease-to-sham ratio (DSR) (**e**). Representative [^68^Ga]Ga-DATA^5m^.SA.FAPi PET/CT scans (coronal view) of the peak of disease activity in the respective model (**f**). Data are expressed as the mean ± SEM (*n* = 3–13 animals/group, * *p* < 0.05); %ID/cc = percentage injected dose per cubic centimeter.

Irrespective of cardiac insult, in vivo imaging (Figure 1a) revealed enhanced uptake of [^68^Ga]Ga-DATA^5m^.SA.FAPi one week post-surgery (MI: 2.4 ± 0.3%ID/cc, SUV_max_ = 0.6 ± 0.1 g/mL (*n* = 5); TAC: 1.9 ± 0.3%ID/cc, SUV_max_ = 0.5 ± 0.1 g/mL (*n* = 3); SHAM: 2.6 ± 0.5 %ID/cc, SUV_max_ = 0.7 ± 0.1 g/mL (*n* = 3)). Two weeks after surgery, the uptake in the MI group tended to increase further (not significant) (2.9 ± 0.3%ID/cc, SUV_max_ = 0.8 ± 0.09 g/mL (*n* = 7)), while TAC uptake remained steady (2.0 ± 0.1%ID/cc, SUV_max_ = 0.6 ± 0.04 g/mL (*n* = 4)), and SHAM hearts showed a slightly decreased uptake (2.1 ± 0.4%ID/cc, SUV_max_ = 0.6 ± 0.1 g/mL (*n* = 3)). Between two and six weeks post-insult, the MI group exhibited a decline in [^68^Ga]Ga-DATA^5m^.SA.FAPi uptake (1.8 ± 0.3%ID/cc, SUV_max_ = 0.5 ± 0.05 g/mL (*n* = 4)), aligning with the SHAM group (1.5 ± 0.1 %ID/cc, SUV_max_ = 0.40 ± 0.04 g/mL (*n* = 3)) by the six-week time point. In contrast, the TAC group showed a slight increase in uptake (2.2 ± 0.3%ID/cc, SUV_max_ = 0.6 ± 0.1 g/mL (*n* = 13)), though this trended downward by the twelfth week (TAC: 1.6 ± 0.3%ID/cc, SUV_max_ = 0.5 ± 0.08 g/mL (*n* = 3)) (Figure 1b,c). The tissue-to-background ratio (TBR) confirmed these results, with a significant difference (*p* = 0.0014) in uptake observed at the one-week time point between TAC and SHAM mice (Figure 1d). Additionally, we analyzed the disease-to-sham ratio (DSR) to further emphasize the disease-specific characteristics. The DSR shows an approximately 50% increase in [^68^Ga]Ga-DATA^5m^.SA.FAPi uptake at the two-week (MI) and six-week (TAC) time points compared to the mean uptake in the sham group (Figure 1e). This increase corresponds to the peak of disease activity in each model and is clearly visualized in the coronal PET/CT images presented in Figure 1f.

### 2.2. Ex Vivo Cardiac Uptake of [^68^Ga]Ga-DATA^5m^.SA.FAPi, Autoradiography and IHC

**Figure 2:**
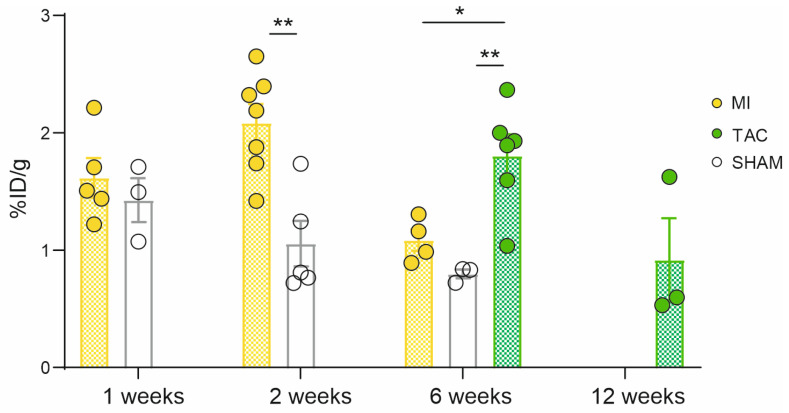
Ex vivo [^68^Ga]Ga-DATA^5m^.SA.FAPi quantification of MI, TAC, and SHAM (control) hearts. Cardiac [^68^Ga]Ga-DATA^5m^.SA.FAPi uptake of MI, TAC, and SHAM mice 1, 2, 6, and 12 weeks post-surgery. Data are expressed as the mean ± SEM (*n* = 3–7 animals/group, * *p* < 0.05, ** *p* < 0.01); %ID/g = percentage injected dose per gram tissue. MI: myocardial infarction; TAC: transverse aortic constriction.

One week after surgery, an enhanced cardiac tracer uptake of [^68^Ga]Ga-DATA^5m^.SA.FAPi was observed (MI: 1.6 ± 0.17%ID/g (*n* = 5); SHAM: 1.4 ± 0.19%ID/g (*n* = 3)). Uptake significantly (*p* = 0.002) increased two weeks after MI induction (2.1 ± 0.16%ID/g (*n* = 7)) compared to SHAM mice (1.1 ± 0.2 %ID/g (*n* = 5)). Six weeks post-surgery, uptake in MI hearts decreased (1.1 ± 0.1%ID/g (*n* = 4)), aligning with the SHAM group (0.8 ± 0.04 %ID/g (*n* = 3)). In contrast, at this time point, heart uptake of the TAC group (1.8 ± 0.2%ID/g (*n* = 6)) was significantly increased compared to both control mice (*p* = 0.007) and the MI group (*p* = 0.018). By twelve weeks after ligation of transverse aorta, cardiac uptake in the TAC group decreased again (0.92 ± 0.35%ID/g (*n* = 3)) (Figure 2). These results confirm the observed in vivo uptake pattern.

In vivo and corresponding ex vivo [^68^Ga]Ga-DATA^5m^.SA.FAPi uptake results are summarized in Table 1, which also includes Cohen’s *d* values in order to indicate the effect size. A medium (*d* ≥ 0.5) to large (*d* ≥ 0.8) effect size could be determined.

**Figure 3:**
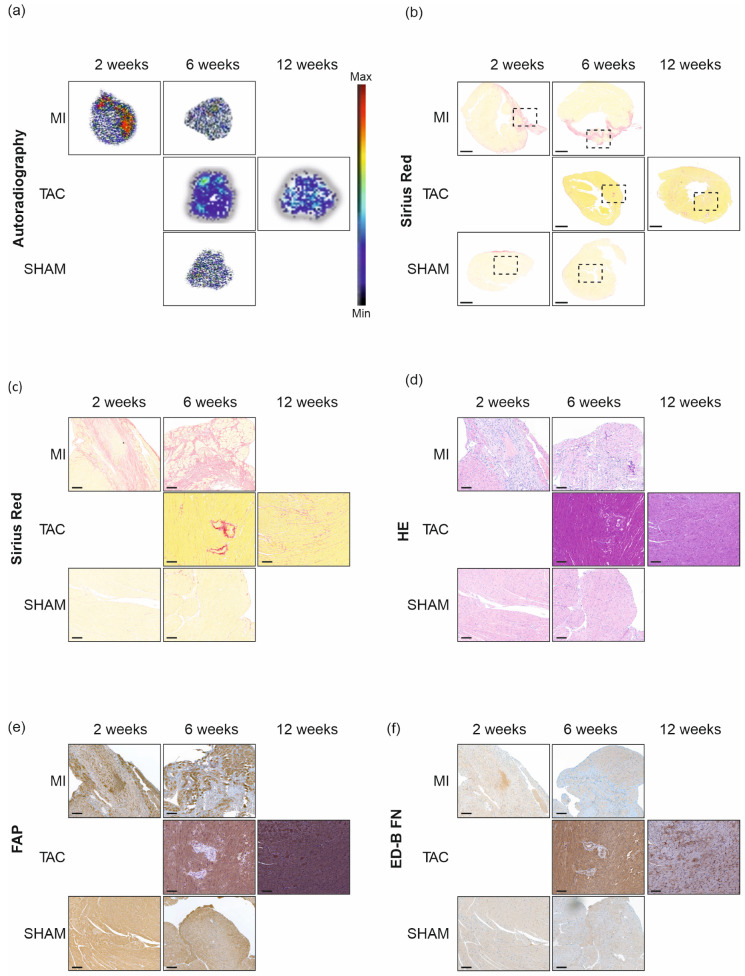
Ex vivo autoradiography, histology, and IHC of MI, TAC, and SHAM (control) hearts. [^68^Ga]Ga-DATA^5m^.SA.FAPi autoradiography (**a**), collagen staining (Sirius Red) (**b**,**c**), Hematoxylin/Eosin (HE) (**d**), FAP (**e**), and ED-B FN (**f**) of representative hearts from MI, TAC, and SHAM mice 2, 6, and 12 weeks post-surgery. The area outlined by the dotted frame (**b**) indicates the selected representative section, which is shown in the subsequent stainings. In (**b**), a magnification factor of 0.8 was selected, which corresponds to 1.00 mm in the size bar shown. In (**c**–**f**), a magnification factor of 10 was selected, which corresponds to 0.100 mm in the size bar shown. MI: myocardial infarction; TAC: transverse aortic constriction.

Ex vivo autoradiography revealed distinct uptake in the area of myocardial infarction (MI) two weeks post-surgery. Consistent with the imaging and biodistribution findings by six weeks, the distribution of [^68^Ga]Ga-DATA^5m^.SA.FAPi was homogeneous, similar to that observed in SHAM mice. Tracer uptake in TAC hearts was also uniformly distributed at both six and twelve weeks (Figure 3a). Histological staining (Sirius Red and Hematoxylin/Eosin (HE)) confirmed the presence of fibrosis (Figure 3b,d). Prominent collagen deposition, as indicated by Sirius Red staining, was observed in the myocardial infarction (MI) area. SHAM hearts displayed collagen on the surface at two weeks post-surgery, but it was undetectable by six weeks. TAC hearts exhibited a homogeneous distribution of collagen (Figure 3b,c). IHC confirmed FAP expression in both diseases, with the highest FAP expression (intensive brown areas) observed in TAC mice 6 weeks post-surgery (Figure 3e). In contrast, the highest ED-B FN expression was detected in TAC mice 12 weeks post-surgery (dark brown areas) (Figure 3f).

### 2.3. Disease Characteristics

**Figure 4:**
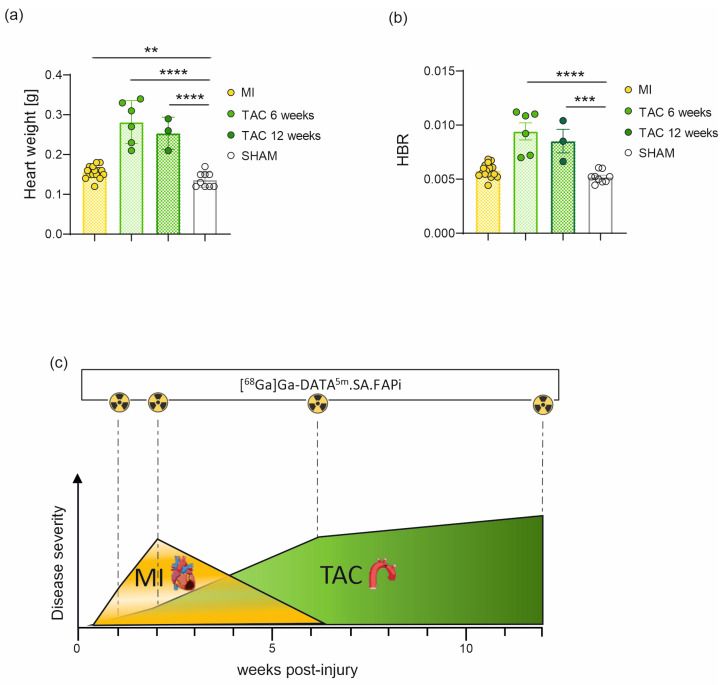
Disease characteristics: Quantitative analysis of heart weight (**a**) and heart-to-bodyweight ratio (HBR) (**b**). Comparison of disease progression and severity as well as imaging time points of MI and TAC mice (**c**). Data are expressed as the mean ± SEM (*n* = 3–16 animals/group, ** *p* < 0.01, *** *p* < 0.001, **** *p* < 0.0001). As no significant differences of the weight values from MI and SHAM hearts were found between the post-surgical time points, values were merged. MI: myocardial infarction; TAC: transverse aortic constriction.

Heart weights of MI mice were significantly (*p* = 0.0096) increased in comparison to the SHAM group one, two and six weeks post-surgery (mean weight across all time points: MI: 0.16 ± 0.004 g; SHAM: 0.14 ± 0.006 g). Additionally, heart weights of the TAC mice were highly significantly (*p* < 0.0001) increased at six (0.28 ± 0.05 g) and twelve weeks (0.25 ± 0.04 g) post-surgery (Figure 4a). The heart-to-bodyweight ratio (HBR) supported this trend, though no significant difference was observed between MI and SHAM groups (Figure 4b). Disease progression revealed distinct peaks in disease severity. Disease peak, corresponding to the maximum tracer uptake, could be observed at the two-week time point for MI mice. This peak is shifted to the six-week time point in case of TAC mice (ongoing interstitial fibrosis), whereas scar formation was already advanced at the same time point for the MI cohort (Figure 4c).

### 2.4. In Vivo [^68^Ga]Ga-DATA^5m^.SA.FAPi Cell Uptake

**Figure 5:**
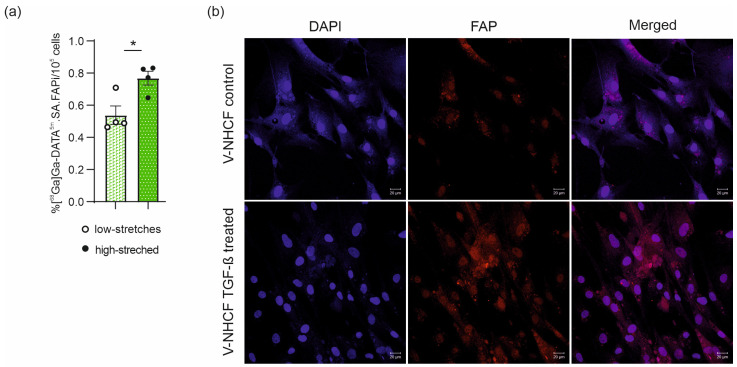
In vitro cell analyses. (**a**) [^68^Ga]Ga-DATA^5m^.SA.FAPi uptake under high- and low-stretched conditions in ventricular human cardiac fibroblasts. Data are expressed as the mean ± SEM (*n* = 4 individual experiments/group, * *p* < 0.05). (**b**) FAP expression in control (top row) and TGF-β treated (bottom row) V-NHCF cells. Representative photomicrographs of nuclei immunostaining using DAPI (left column), FAP staining (middle column), and the merged stainings (right column). The white line represents the scale and corresponds to 20 µm. Control V-NHCF cells (*n* = 3) and V-NHCF TGF-β treated (20 ng/mL) for 24 h (*n* = 3). V-NHCF: ventricular normal human cardiac fibroblasts.

In order to clarify whether the increase of [^68^Ga]Ga-DATA^5m^.SA.FAPi uptake in the myocardium resulted from cardiac fibroblast, ventricular normal human cardiac fibroblasts (V-NHCF) were used and exposed to high-stretching condition, reflecting the pressure overload hypertrophy. High-stretched condition resulted a significantly (*p* = 0.0180; *n* = 4) increased uptake (0.769 ± 0.043%/10^5^ cells) compared to low stretched cells (0.538 ± 0.057%/10^5^ cells) (Figure 5a). In line with that, FAP staining intensity was enhanced in V-NHCF subjected to TGF-β treatment (Figure 5b).

### 2.5. Clinical [^68^Ga]Ga-DATA^5m^.SA.FAPi PET/CT

**Figure 6:**
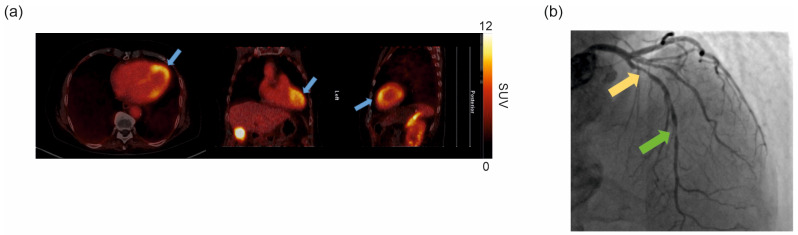
Representative clinical cardiac images: PET/CT images performed 120 min after i.v. tracer injection. Enhanced [^68^Ga]Ga-DATA^5m^.SA.FAPi uptake is observed in the mid-distal anterior LV segments in axial (left), coronal (middle), and sagittal (right) views (blue arrows) (**a**). Coronary angiography image: Right anterior oblique cranial view shows the left coronary artery system with a significant stenosis on the proximal LAD coronary artery (yellow arrow). A further significant stenosis with subtotal occlusion can be observed on the mid-LAD coronary artery (green arrow) (**b**).

LV [^68^Ga]Ga-DATA^5m^.SA.FAPi uptake on PET/CT images is depicted in Figure 6a. This patient presents an enhanced [^68^Ga]Ga-DATA^5m^.SA.FAPi uptake in the mid-distal anterior LV segments (SUV_max_ = 11.6 g/mL; TBR = 4). Angiographic imaging identified a coronary artery disease of the left coronary system demonstrating the presence of significant stenosis on the proximal left anterior descending (LAD) coronary artery as well as a subtotal occlusion on the mid-LAD coronary artery (Figure 6b).

## 3. Discussion

To the best of our knowledge, this study is the first to evaluate the potential of [^68^Ga]Ga-DATA^5m^.SA.FAPi for imaging of the fibrotic process in response to MI and pressure overload insult. Previous studies demonstrated the significantly increased cardiac uptake of [^68^Ga]Ga-FAPI-04 in murine models of ischemic [2] and non-ischemic heart failure [17]. Here, we aimed to detect the molecular sign for cardiac fibrosis in those models of replacement or interstitial fibrosis using [^68^Ga]Ga-DATA^5m^.SA.FAPi.

Cardiac fibrosis is a common pathophysiological phenomenon in patients with left ventricular hypertrophy, pulmonary artery hypertension and dilated cardiomyopathies. Pathological remodeling of the ECM and cardiac fibrosis leads to a progressive deterioration of systo-diastolic properties mainly due to a stiffened myocardial matrix. The pathways governing pathological ECM remodeling are not fully elucidated and, most importantly, breakthrough anti-fibrotic therapies have yet to be approved. Therefore, both targeting and visualizing ECM remodeling at an early time point represent a major therapeutic goal to reduce the burden of cardiovascular diseases. FAP plays an important role in cardiac wound healing and remodeling. Targeting FAP with specific tracers could provide insights into disease development and progression. Hence, FAPI tracers also could be used as novel molecular theranostic ligands for a variety of cardiomyopathies [3,8,9].

In our study, preclinical PET/CT scans were performed 1, 2, 6, and 12 weeks post-surgery (Figure 1a–d). The maturation of scar tissue due to the massive collagen deposition, predominantly released by the myofibroblasts, shows a spatial and temporal pattern following MI [30]. We found a significant upregulation of cardiac uptake of [^68^Ga]Ga-DATA^5m^.SA.FAPi two weeks after the MI insult, which was attenuated after six weeks, demonstrating that replacement fibrosis and infarct healing was completed. This is in line with the scar formation (collagen cross-linking), which is largely completed in mice 35–42 days after the MI insult. Notably, the highest tracer uptake in SHAM mice could be observed one week after surgery, with levels similar to the MI group. That may be attributed to the initiation of FAP expression due to general wound healing after surgery.

The replacement form of cardiac fibrosis responds to cardiomyocyte death, e.g., after MI, while other forms of cardiac fibrosis such as perivascular or interstitial fibrosis are not related to cardiomyocyte necrosis. Interstitial cardiac fibrosis is a hallmark of pressure overload induced hypertrophy, and its presence is associated with worse clinical outcome [31]. Therefore, it is urgent to find potential markers for longitudinal monitoring of cardiac interstitial fibrosis in patients among hypertrophic cardiomyopathy, aortic valve stenosis, and untreated peripheral hypertension. Indeed, cardiac echocardiography and magnetic resonance imaging (MRI) are useful tools for comprehensive functional and morphological assessment of the heart, but they do not fulfil the request to detect and monitor the process of cardiac fibrosis at the molecular levels. Therefore, we investigated [^68^Ga]Ga-DATA^5m^.SA.FAPi uptake in interstitial fibrosis in the pressure overload heart-induced cardiac hypertrophy group (TAC). TBR was significantly increased compared to the SHAM group one week post-surgery and the uptake increased steadily up to six weeks after surgery due to the progression of fibrosis. By the final time point, twelve weeks after the constriction of the transverse aorta, measurements showed a decreased cardiac uptake of [^68^Ga]Ga-DATA^5m^.SA.FAPi. This reduction may be attributed to a downregulation of FAP in this stage of LV hypertrophy, alongside an associated upregulation of ED-B FN, as confirmed by IHC (Figure 3e,f).

Ex vivo biodistribution analysis (Figure 2) corroborated the imaging outcomes, revealing a significantly increased cardiac uptake of [^68^Ga]Ga-DATA^5m^.SA.FAPi in MI mice two weeks after surgery and in TAC mice six weeks post-surgery. Consistent with the in vivo imaging data, SHAM mice exhibited the highest uptake at the early stage. TAC mice were used for longitudinal imaging measurements; hence, ex vivo data were not available for the one- and two-week time points. The observed disparities in heart weight between the MI, SHAM, and TAC mouse models are closely associated with the different disease progression of myocardial infarction and pressure overload heart failure, as evidenced by the heart-to-bodyweight ratio (Figure 4a–c).

IHC (Figure 3) revealed increased FAP expression in the MI group, with FAP being strictly localized to the area of myocardial infarction. FAP could not be detected in the surrounding granulation tissue during remodeling. In contrast, FAP expression could be detected in the entire heart of mice in the TAC group. The highest FAP expression was observed six weeks after surgery, whereas the highest expression of ED-B FN could be found at twelve weeks post-surgery. These results might provide a possible explanation for the reduced tracer uptake at the latter time point. The temporal but also the spatial distribution of FAP was demonstrated in the two disease models.

Ex vivo autoradiography confirmed the IHC finding, showing increased uptake in the infarction area compared to the control group, where the overall uptake was decreased and homogenously distributed. In addition, autoradiography also demonstrated a uniformly distributed uptake of [^68^Ga]Ga-DATA^5m^.SA.FAPi in TAC mice. This may reflect this type of heart failure, where collagen and FAP expression affects the heart in its entirety. The lack of autoradiography of SHAM mice two weeks post-surgery is caused by the short half-life of gallium-68, which made it difficult to obtain valuable data of all animals. Another limitation of our study was that we did not include a control cohort for the twelve weeks post-surgery time point due to the limited amount of precursor.

PET imaging was not able to identify the cellular source of FAP uptake in the myocardium. However, evidence suggests that myofibroblasts largely express FAP. Therefore, we next investigated whether [^68^Ga]Ga-DATA^5m^.SA.FAPi uptake was changed in cardiac fibroblasts, which were exposed to an extensive stretch protocol. The results revealed significant disparities between low- and high-stretched levels (Figure 5), which also correlate with the findings from the later stages of the TAC mouse model.

In the clinical scan, prominent [^68^Ga]Ga-DATA^5m^.SA.FAPi uptake was observed in the mid-distal anterior LV. While cardiac amyloidosis could not be confirmed via LGE on CMR, coronary angiography identified significant stenosis on the proximal and mid-LAD coronary artery (Figure 6). These findings suggest that [^68^Ga]Ga-DATA^5m^.SA.FAPi uptake may be associated to myofibroblasts activation (due to insufficient vascularization) in the mid-distal anterior LV wall and could serve as a potential imaging marker for predicting future LV cardiac remodeling.

Overall, our results consistently revealed a distinct uptake pattern that clearly reflected disease progression. However, [^68^Ga]Ga-DATA^5m^.SA.FAPi exhibited a higher background signal compared to other FAPI tracers. The elevated background signal could potentially explain the loss of statistical significance observed in imaging data compared to biodistribution measurements. Nevertheless, [^68^Ga]Ga-DATA^5m^.SA.FAPi demonstrated comparable results to well-established FAPI-PET-tracers, offering a viable alternative for the imaging of cardiac fibrosis.

Recent studies have reported increased FAPI tracer uptake in diabetic heart injury [32], as well as in both acute and chronic myocardial infarction [33,34]. Moreover, Chen et al. [35] demonstrated that [^18^F]AlF-FAPI could detect cardiac fibrosis and predict left ventricular mechanical dyssynchrony in patients with coronary artery disease and degenerative mitral valve regurgitation.

In addition, our in vitro experiments using human ventricular cardiac fibroblasts provide a novel and cost-effective approach to evaluate the potential of FAPI tracers for cardiac imaging. Collectively, these findings underscore the growing relevance of FAP inhibitor tracers in monitoring the progression of cardiac fibrosis and potentially guiding therapy in patients with various forms of heart failure.

## 4. Materials and Methods

### 4.1. Radiosynthesis of [^68^Ga]Ga-DATA^5m^.SA.FAPi

Unless otherwise stated, the chemicals used were purchased from Sigma Aldrich (St. Louis, MO, USA). For this study, the precursor was produced according to Moon et al. [4]. Before labelling DATA^5m^.SA.FAPi with gallium-68, 300 μL of 1M NH_4_OAc buffer (pH 5.5) was added. Subsequently, a ^68^Ge/^68^Ga-generator (IRE *Galli Eo*^®^, Fleurus, Belgium) was eluted with a 0.1 M HCl solution. The precursor compound was added to the activity and incubated on a dry block heater (IKA^®^, Staufen, Germany) at 50 °C for 10 min. For separation of free gallium-68 and colloid a SEP-PAK C18 light cartridge (Waters Corporation, Milford, MA, USA; preconditioned with ethanol and water) was used and the product was obtained by elution with 1 mL of 1:1 ethanol/water. In order to adjust osmolality and pH required for an intravenous (i.v.) injection, 2 mL of a physiological saline solution (0.9%, Braun, Melsungen, Germany) was added to the product. With the available amount of precursor, a total of 38 successful syntheses could be performed. The required purity was ensured by using radio-HPLC and radio-TLC. The latter was conducted on iTLC-SG plates (Agilent, Santa Clara, CA, USA) with either 1 M citrate buffer (pH 4) or 1M NH_4_OAc (NH_4_OAc for analysis, EMSURE^®^, Merck KGaA, Darmstadt, Germany) buffer (pH 5.5) combined with DMF (N,N Dimethylformamide 99.8%, ExtraDry, AcroSeal™, Thermo Fisher Scientific, Waltham, MA, USA) 1:2. Both buffers were adjusted with HCl (Reag.Ph.Eur. 0.1 mol/L, Carl Roth GmbH, Karlsruhe, Germany) TLCs were measured via a TLC imager (Elysia Raytest GITA*, Straubenhardt, Germany). Analysis with Gina Star TLC 6.3 (Elysia-Raytest. Straubenhardt, Germany) revealed an Rf value of 0.1–0.2 for the product and 0.7–0.9 for free radio metal for the citrate buffer, whereas the Rf values for the product amounted to 0.55–0.75 and 0.02–0.04 for colloid for the NH_4_OAc buffer. The analytical HPLC (VWR HITACHI Chromaster, Tokio, Japan) was conducted with a Chromolith^®^ Preformance RP-18e (100–4.6 mm) column (Merck KGaA, Darmstadt, Germany) and a linear gradient of 5–95% MeCN (+0.1% TFA)/95–5% purified water (+0.1% TFA) over 10 min. The chromatography was analyzed with the software CLARITY 7.3 (Data Apex, Prague, Czech Republic) and resulted in a purity of 98.5 ± 1.4% (*n* = 38).

### 4.2. Surgical Mouse Models

Male C57BL/6J mice provided by Charles River Laboratories of an age of 10 to 12 weeks were used. Animals were housed under standardized conditions with a 12 h day-night cycle and ad libitum standard diet. The experimental protocol was approved by the intramural animal ethics committee at the Medical University of Vienna and the Austrian Ministry of Education, Science and Research (BMBWF-66.009/0224-V/3b/2019; date of study approval: 3 July 2019), conforming with the Guide for the Care and Use of Laboratory Animals published by the US National Institutes of Health (NIH Publication No. 85–23, revised 1996).

### 4.3. General Anesthesia, Analgesia, and Preparation

Mice were anesthetized by injection of medetomidine, midazolam, fentanyl, and ketamine (MMFK). After intubation mechanical ventilation was ensured with a respiratory rate of 150 per minute. Additionally, 1–1.5% isoflurane was used to ensure sufficient anesthesia depth throughout the whole procedure. The mice were placed in a supine position on a heating plate and their extremities were fixed. To prevent dehydration of the eyes, ointment was applied. The surgery was performed under constant ECG surveillance as well as monitoring of respiratory rate and body temperature.

### 4.4. Mouse Model of Myocardial Infarction

Myocardial infarction (MI) was induced surgically as described previously [36]. Mice were placed in a right lateral position and thoracotomy was performed at the fourth intercostal space. After removal of the pericardium, the left coronary artery was permanently occluded using a 7–0 monofilament suture (PROLENE^®^, Ethicon, LLC., San Lorenzo, PR, USA). MI was confirmed visually by paling of the infarct region and ST elevation on the ECG. After closing thorax and skin, mice were injected subcutaneous (s.c.) with atipamezole and flumazenil to antagonize MMFK. Sham-operated mice underwent an identical surgical procedure, but without ligation of the coronary artery.

### 4.5. Pressure Overload Induced LV Hypertrophy Model

In order to initiate pressure overload LV hypertrophy and concomitant cardiac fibrosis, transverse aortic constriction (TAC) was performed as described previously [37]. The mice were placed in a supine position on a heating plate and their extremities were fixed. The chest was opened via a median sternotomy starting cranially until the second rib. The thymus was dissected to provide access to the aortic arch. The ascending aorta was visualized by dissecting thymus and perivascular fat tissue. A 6–0 silk suture (PERMAHAND^®^, Ethicon, LLC., San Lorenzo, PR, USA) was pulled under the ascending aorta between the innominate artery and left common carotid artery. A spacer (27G needle) was placed below the suture to avoid the complete constriction of the aorta. After fixation of the suture, the spacer was removed. The chest and skin were closed again, and mice were injected s.c. with atipamezole and flumazenil to antagonize MMFK. Sham-operated mice underwent the identical surgical procedure, without constriction of the aorta.

### 4.6. Postoperative Care

To support postoperative recovery, all animals received a single s.c. injection of 5% glucose solution. Mice were extubated when spontaneous respiration was detectable. For additional postoperative analgesia, mice received a single intraperitoneal (i.p.) injection of buprenorphine; mice also received piritramide through the drinking water for the first three days postoperatively.

### 4.7. Preclinical In Vivo Imaging of [^68^Ga]Ga-DATA^5m^.SA.FAPi and Data Analysis

For imaging experiments, different post-surgical time points were investigated (MI/SHAM: 1, 2, and 6 weeks; TAC: 1, 2, 6, and 12 weeks; Table 2). Mice were injected i.v. over the lateral tail vein with 15–25 MBq [^68^Ga]Ga-DATA^5m^.SA.FAPi (in 150–250 μL) under isoflurane (2–3% in oxygen). After 45 min distribution under anesthesia, a 10 min static PET scan followed by a 5 min CT scan was conducted on a Siemens Inveon^®^ Multi-Modality microPET/CT Scanner (Siemens Healthineers, Erlangen, Germany). Subsequently, after the PET/CT scan, mice needed for longitudinal imaging were taken out of anesthesia, and the other animals were sacrificed via cervical dislocation for further processing. The CT raw data were reconstructed with an OSEM algorithm followed by standard mouse beam-hardening correction and noise reduction (matrix size: 1024 × 1024; effective pixel size: 97.56 μm). The dedicated CT image data were calibrated to Hounsfield Units (HU). PET list mode data were sorted into three-dimensional sinograms and reconstructed using a Feldkamp algorithm without scatter correction but using a ramp filter (matrix size 256 × 256). The data were normalized and corrected for random, dead time, and radioactive decay. A calibration factor was applied to convert the activity information into absolute concentration units.

Multimodal (PET/CT) rigid-body image registration and biomedical image quantification was performed using the image analysis software PMOD 3.8 (PMOD Technologies, Zürich, Switzerland). The CT images were fused to the respective PET scans followed by creating volumes of interest (VOIs). The tracer uptake in the VOIs was normalized to injected dose and animal weight. To enable comparability of the results between the animals, values are expressed as percent of the injected dose per cubic centimeter (%ID/cc), as well as the maximum standardized uptake values (SUV_max_; g/mL). For calculating the tissue-to-background ratio (TBR), the blood pool was used as the background in both the MI and SHAM groups, whereas the triceps brachii muscle served as the background in the TAC group. This selection was based on the need to account for physiological differences in each model, where the blood pool is a more stable reference in MI and SHAM groups, while muscle tissue, such as the triceps brachii, provides a more relevant background in the TAC group due to the distinct perfusion characteristics. Disease-to-sham ratio (DSR) was normalized to the mean SUV_max_ value of the respective SHAM cohort.

### 4.8. Ex Vivo Biodistribution and Autoradiography

For subsequent biodistribution, hearts were harvested, weighed, and stored on crashed ice for further processing. Their radioactivity was measured in a gamma counter (2480 Automatic Gamma Counter Wizard^2^ 3”, Perkin Elmer, Waltham, MA, USA). After decay correction, results were expressed as percent of the injected dose of radioactivity per gram of tissue (%ID/g). After measurement in the gamma counter, the heart was divided into two parts: one part was harvested for histology and IHC. The other part was frozen in liquid nitrogen and embedded in Optimal Cutting Temperature (OCT) compound (Tissue-Tek^®^, Sakura Finetek Europe B.V.) for slicing. The organ was sectioned into 20 to 30 μm thin slices, using a cryostat (Epredia CryoStar NX70, Kalamazoo, MI, USA) and mounted on microscope slides (Menzel Gläser, Superfrost^®^ Plus, Braunschweig, Germany). Subsequently, all slides were placed on a multisensitive storage phosphor film (Multisensitive Phosphor Screens Long Type MS, PPN 7001724, Perkin Elmer, Waltham, MA, USA) and stored in a light- and radiation-shielded cassette overnight [38]. The analysis of the film was carried out with a Phosphor Imager (Perkin Elmer Cyclone^®^ Plus, Waltham, MA, USA) or (Elysia Raytest CR 35 Bio Plus, Straubenhardt, Germany) and a dedicated software (Elysia-Raytest AIDA Image Analysis Software 5.1, Straubenhardt, Germany).

### 4.9. Histology and Immunohistochemistry

IHC, hematoxylin/eosin (HE), and Sirius Red (for collagen) stainings were performed with formalin-fixed paraffin-embedded (FFPE) heart tissues, using consecutive sections (3–5 µm). IHC was performed for FAP ( PA5-99313, Thermo Fisher Scientific, Waltham, MA, USA) and extra-domain B fibronectin (ED-B FN) (ED-B Affilin^®^, Navigo Proteins GmbH, Halle, Germany). IHC stainings were performed following standard protocols. ED-B Affilin^®^ immunostaining had an additional Strep-Tag (GenScript, A00626, Leiden, The Netherlands) step. All samples were scanned with the Ventana DP200 (Roche, Basel, Switzerland) and processed with the Image Viewer MFC Application. Images were analyzed based on optical observation.

### 4.10. Human Cardiac Fibroblasts Cell Culture

Ventricular Normal Human Cardiac Fibroblasts (V-NHCFs) from Lonza© (Lonza Group AG, Basel, Switzerland) were cultured as described previously [39]. V-NHCFs were cultivated with Fibroblast Growth Medium-3 (FGM-3) with the supplements 10% Fetal Bovine Serum (FBS), 0.1% Gentamicin Sulphate-Amphotericin (GA-1000, Lonza), 0.1% Insulin, 0.1% human fibroblast growth factor (hFGF), and 0.1% Insulin-like growth factor (R3-IGF1) in an incubator at 37 °C with 5% CO_2_. The cells were cultured in T175 cell culture flasks (Lonza) under sterile conditions, using a laminar flow hood.

V-NHCFs (approximately 200.000) were seeded onto 6-well FlexCell-plates with Collagen 1 coating (Bioflex plates Collagen 1, Flexcell International Corporation, Burlington, NC, USA) under sterile conditions. The cells were kept in an incubator (HeraCell 150i, Thermo Fisher Scientific, Waltham, MA, USA) with 37 °C and 5% CO_2_. After checking and emptying the water containers, the cell seeder plates (25 mm) were added onto the baseplate. The 6-well plates were put into silicone sealings and added onto the cell seeders. After 24 h of seeding, the cells were checked under the microscope and then additional 1 mL of cell culture media was added onto the cells. The cells were loaded under the same airtight conditions and 24 h of high elongation regime (18–21% elongation and approximately −75 kPa pressure) or low elongation regime (1–3% elongation and approximately −7 kPa pressure), respectively. After one of the regimes was completed, the cells were used for in vitro [^68^Ga]Ga-DATA^5m^.SA.FAPi cell uptake assessment.

### 4.11. In Vivo [^68^Ga]Ga-DATA^5m^.SA.FAPi Cell Uptake and FAP Expression

A 90 µL cell suspension, containing 10^5^ fibroblasts with different treatments (high and low stretched method), was incubated in 96-well filter plates (MADVN6550, Merck Millipore, Darmstadt, Germany) with 60 µL of a 0.4 MBq/mL tracer solution. To assess unspecific binding, the filter plates alone were incubated with the same [^68^Ga]Ga-DATA^5m^.SA.FAPi concentration under identical conditions (blank). After 60 min of incubation at 37 °C, the cells were rinsed off with PBS (Gibco, Thermo Fisher Scientific, Waltham, MA, USA) (2  ×  100 µL) by vacuum filtration through the plate. The filters containing the cells were individually transferred into tubes, using a commercial punch kit (MAMP09608, Merck Millipore, Darmstadt, Germany) and measured in a gamma counter. Additionally, 60 µL of the 0.4 MBq/mL radiotracer solution was separately measured in the gamma counter to quantify the maximum amount of added radioactivity/well (100% [^68^Ga]Ga-DATA^5m^.SA.FAPi). The uptake was quantified as percentage of added radioactivity per 10^5^ cells. The uptake of the blank was less than 0.1%.

V-NHCF cells were cultivated identically as described above. Cells (approximately 40,000) were seeded onto a 24-well plate in 22 mm diameter glass coverslips under sterile conditions. The cells were kept in an incubator (HeraCell 150i, Thermo Fisher Scientific, Waltham, MA, USA) at 37 °C and 5% CO_2_; 24 h after seeding, three replicates were treated with 20 ng/mL TGF-β (Recombinant human TGF-β1, 240-B/CF, Bio-Techne, R&D Systems, Minneapolis, MN, USA) for 24 h and three replicates were used as the control group. After that, we performed the immunocytochemistry for FAP; therefore, cells were washed with 1x PBS, fixed in 4% paraformaldehyde in PBS for 20 min at RT, and again washed with PBS. The coverslips were incubated with 100 mM glycine in PBS for 15 min and washed 3× with PBS for 5 min, and the cells were permeabilized in PBS containing 0.1% Triton X-100 for 5 min.

For blocking of unspecific binding sites, the coverslips were incubated with 4% BSA in PBS for 1h at RT. Primary antibody dilutions were prepared in PBS (FAP, 1:200, PA5-99313, Thermo Fisher Scientific, Waltham, MA, USA), and the coverslips were incubated with antibody solution for 1h at RT. Afterwards, the coverslips were washed 3× with PBS for 5 min. Fluorescence-labelled secondary antibody (Texas Red TI-1000, 1:200, VectorLabs, Newark, CA, USA), diluted in PBS, was added and incubated for 1 h at RT under light protection in the dark. Coverslips were washed 3x with PBS for 5 min and mounted by placing them onto a small drop of Mowiol with DAPI on a glass slide. Samples were dried overnight at RT in the dark and pictures were afterwards captured with a Leica Microscope (LSM700, Wetzlar, Germany).

### 4.12. Clinical [^68^Ga]Ga-DATA^5m^.SA.FAPi PET/CT Study

As parts of a clinical study (Ethical Approval no. 1400/2020; date of study approval: 26 May 2020), patients with severe aortic stenosis underwent [^68^Ga]Ga-DATA^5m^.SA.FAPi PET/CT and cardiac magnetic resonance (CMR) imaging (1.5T CMR scanner, Avanto Fit, Siemens Healthineers, Erlangen, Germany) to investigate the potential presence of AL cardiac amyloidosis. In this report, we included a PET/CT scan of one patient from the cohort. The scan of the heart region was performed 120 min after injection of the tracer (~180 MBq) with a PET acquisition time of 15 min and a low-dose CT for attenuation correction (Siemens Biograph True Point 64 PET/CT, Siemens Healthineers, Erlangen, Germany). For visual analysis, CT and PET images were matched and fused into transaxial and coronal images of 5 mm thickness. SUV_max_ and TBR (blood pool was used as background) were calculated. On the same day, the late gadolinium enhancement (LGE) CMR images were obtained for advanced tissue characterization according to current protocol recommendations [40]. For contrast and LGE acquisitions, 0.15 mmol/kg Gadovist contrast medium was injected. Additionally, a coronary angiography was conducted.

### 4.13. Statistical Analysis

Statistical analyses were performed in GraphPad Prism 8.2.1 (GraphPad Software, Inc., San Diego, CA, USA). Values are represented as arithmetic means ± standard error mean (SEM). Statistical significance was calculated with multiple/unpaired *t*-test when comparing two groups or with two-way ANOVA test when comparing more than two groups. Significance is indicated in the figures by *: *p*-value < 0.05; **: *p*-value < 0.01; ***: *p*-value < 0.001; ****: *p*-value < 0.0001. To assess the effect size between groups, Cohen’s *d* values were calculated. *d* values around 0.2 were considered small, 0.5 medium, and 0.8 or greater large effect sizes.

## 5. Conclusions

In summary, our data confirm and extend prior research, reaffirming the potential of FAPI-PET tracers for imaging fibrotic diseases. Our results revealed the spatial and temporal distribution pattern of [^68^Ga]Ga-DATA^5m^.SA.FAPi uptake in distinct forms of cardiac fibrosis. Additionally, our in vitro experiments with human ventricular cardiac fibroblasts may represent a novel, cost-effective approach to validate FAPI tracers. Collectively, these results further emphasize the value of FAP inhibitor tracers for monitoring cardiac fibrosis progression and guiding therapy for patients suffering from different entities of heart failure.

## Figures and Tables

**Table 1:** In vivo and ex vivo results at all measurement time points.

		In Vivo			Ex Vivo		
Time Point	Mouse Model	SUV_max_ [g/mL]	*n*	Cohen’s d	%ID/g	*n*	Cohen’s d
**1** **week**	MI	0.6 ± 0.1	5	0.068	1.6 ± 0.17	5	0.544
	TAC	0.5 ± 0.1	3	0.730			
	SHAM	0.7 ± 0.1	3		1.4 ± 0.19	3	
**2** **weeks**	MI	0.8 ± 0.09	7	0.983	2.1 ± 0.16	7	2.392
	TAC	0.6 ± 0.04	4	0.113			
	SHAM	0.6 ± 0.1			1.1 ± 0.2	5	
**6** **weeks**	MI	0.5 ± 0.05	4	0.861	1.1 ± 0.1	4	2.087
	TAC	0.6 ± 0.1	13	0.918	1.8 ± 0.2	6	3.131
	SHAM	0.4 ± 0.04	3		0.8 ± 0.04	3	
**12 weeks**	MI						
	TAC	0.5 ± 0.08	3		0.9 ± 0.35	3	
	SHAM						

**Table 2:** Number of mice in each group at the respective PET scan time points and ex vivo biodistribution (biodis) analysis.

	1 Week		2 Weeks		6 Weeks		12 Weeks	
Mouse Model	Scan	Biodis	Scan	Biodis	Scan	Biodis	Scan	Biodis
**MI**	5	5	7	7	4	4		
**SHAM**	3	3	5	5	3	3		
**TAC**	3		4		13	6	3	3

## Data Availability

The datasets generated during and/or analyzed during the current study are available from the corresponding author on reasonable request.

## Abbreviations

The following abbreviations are used in this manuscript:

AL	amyloid light chain
CAF	cancer associated fibroblast
CMR	cardiovascular magnetic resonance
CT	computed tomography
DATA	2,2′-(6-((carboxymethyl)amino)-1,4-diazepane-1,4-diyl) diacetic acid
DOTA	1,4,7,10-Tetraazacyclododecane-1,4,7,10-tetraacetic acid
DSR	disease-to-sham ratio
EDTA	ethylenediaminetetraacetic acid
ECM	extracellular matrix
FAP	fibroblast activation protein
FAPi	FAP inhibitor
FBS	fetal bovine serum
HBR	heart-to-bodyweight ratio
HCl	hydrochloric acid
HE	hematoxylin/eosin
HPLC	high-performance liquid chromatography HU: Hounsfield Units
IHC	immunohistochemistry
i.p.	intraperitoneal
i.v.	intravenous
LAD	left anterior descending coronary artery
LGE	late gadolinium enhancement
LV	left ventricular
MI	myocardial infarction
MeCN	acetonitrile
MRI	magnetic resonance imaging
NH4OAc	ammonium acetate
PBS	phosphate buffered saline
PET	positron emission tomography
SA	squaric acid
s.c.	subcutaneous
TAC	Transverse aortic constriction
TBR	tumor-to-background ratio
TLC	thin layer chromatography
TME	tumor microenvironment
V-NHCF	ventricular normal human cardiac fibroblasts
